# Functional Redundancy Patterns Reveal Non-Random Assembly Rules in a Species-Rich Marine Assemblage

**DOI:** 10.1371/journal.pone.0026735

**Published:** 2011-10-21

**Authors:** Nicolas Guillemot, Michel Kulbicki, Pascale Chabanet, Laurent Vigliola

**Affiliations:** 1 UR-CoRéUs, IRD (Institut de Recherche pour le Développement), Noumea, New Caledonia; 2 UMR 985 (Agrocampus Ouest, INRA Ecologie et Santé des Ecosystèmes), Université Européenne de Bretagne, Agrocampus Ouest, Rennes, France; 3 Koniambo Nickel SAS, Noumea, New Caledonia; 4 UR-CoRéUs, IRD (Institut de Recherche pour le Développement), Banyuls sur mer, France; 5 UR-CoRéUs, IRD (Institut de Recherche pour le Développement), Ste Clotilde, La Réunion, France; Swansea University, United Kingdom

## Abstract

The relationship between species and the functional diversity of assemblages is fundamental in ecology because it contains key information on functional redundancy, and functionally redundant ecosystems are thought to be more resilient, resistant and stable. However, this relationship is poorly understood and undocumented for species-rich coastal marine ecosystems. Here, we used underwater visual censuses to examine the patterns of functional redundancy for one of the most diverse vertebrate assemblages, the coral reef fishes of New Caledonia, South Pacific. First, we found that the relationship between functional and species diversity displayed a non-asymptotic power-shaped curve, implying that rare functions and species mainly occur in highly diverse assemblages. Second, we showed that the distribution of species amongst possible functions was significantly different from a random distribution up to a threshold of ∼90 species/transect. Redundancy patterns for each function further revealed that some functions displayed fast rates of increase in redundancy at low species diversity, whereas others were only becoming redundant past a certain threshold. This suggested non-random assembly rules and the existence of some primordial functions that would need to be fulfilled in priority so that coral reef fish assemblages can gain a basic ecological structure. Last, we found little effect of habitat on the shape of the functional-species diversity relationship and on the redundancy of functions, although habitat is known to largely determine assemblage characteristics such as species composition, biomass, and abundance. Our study shows that low functional redundancy is characteristic of this highly diverse fish assemblage, and, therefore, that even species-rich ecosystems such as coral reefs may be vulnerable to the removal of a few keystone species.

## Introduction

Diversity is essential to ecosystem functioning [1]–[5]. In particular, the importance of functional diversity has recently been stressed with regard to the long-used taxonomic diversity [6], [2], [7]–[10]. Yet, in most cases, diversity is still measured from lists of species, not functions. This is unfortunate because changes in functional diversity rather than changes in taxonomic composition are likely to affect the stability, resistance and resilience of species assemblages [9], [11].

Whether functions are more important than species or not depends on the extent of functional redundancy, i.e. the number of taxonomically distinct species that exhibit similar ecological functions [12]. Because highly redundant functions are more persistent than constituent species, the functioning of ecosystems with high functional redundancy will necessarily be more affected by the removal of a function than of a species [13]. This has deep implications in conservation ecology. For example, in the case of a disturbance, changes in the type and number of functions may have greater consequences for the ecosystem than changes in taxonomic composition [14], [15]. However, species loss may be equivalent to function loss for ecosystems with low functional redundancy. For these, one species may indeed represent a unique function. In reality, the functioning of natural ecosystems is ensured by a range of functions. Some of these will be highly redundant, whereas others will be ensured by only one or a few species (e.g. top predators). Thus, whether an ecosystem can be considered functionally redundant as a whole, and thus more stable, resistant and resilient, will depend upon the ratio between the number of species and functions. This ratio is not constant; it is a function of species diversity [16]. This implies that ecosystem functioning depends on the strength, shape and nature of the relationship between functional and species diversity. At the moment, this relationship remains poorly understood for nearly all ecosystems [17].

**Figure 1:**
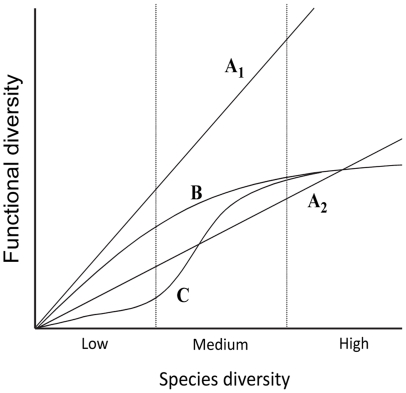
Relationship between taxonomic diversity and functional diversity: schematic scenarios (redrawn from [**16**]).

The relationship between taxonomic and functional diversity is necessarily increasing and going through the origin of the graph. Indeed, adding new species to an assemblage can only increase the number of functions or the redundancy of existing functions. A steep slope in the relationship indicates the fast emergence of new functions, whereas a gentle slope implies a greater redundancy of existing functions. Based on this, [16] proposed four schematic relationships between taxonomic and functional diversity (Figure 1). In the first scenario (A1), each species plays a unique functional role, resulting in a 1∶1 linear relationship between the two types of diversity. All other scenarios assume that multiple species can perform similar functions, i.e. some redundancy exists. The second scenario (A2) is a linear relationship with a <1 slope. It implies that a new function can emerge at a constant incremental rate in species diversity. The third scenario (B) describes ecosystems where functional diversity increases rapidly at low species diversity and subsequently increases at declining rates as and when the number of functions represented in the assemblage becomes important. The last scenario (C) assumes that the relationship between species and functional diversity varies with environmental conditions or habitats. Whereas only a few species sharing a limited set of functional traits can coexist in a simple environment, transition towards a new, more complex environment would be characterised by an abrupt increase in both species and functional diversity. The relationship would then stabilise at values that are characteristic of the second environment/habitat. Typically, this S-shaped scenario would occur when an ecosystem recovers from a disturbance. Both curvilinear scenarios (B and C) may or may not reach an asymptote. If past a given number of species all functional roles are represented, then the relationship between taxonomic and functional diversity will become flat [18]. In this case, it would be critical for both ecology and conservation to determine at what level of diversity this asymptote may occur. Alternatively, a non-asymptotic relationship would imply that only high levels of taxonomic diversity can allow the regular installation of new species with unique and probably rare functions [19], [20]. In this case, a small level of environmental degradation on a large scale may quickly make these species endangered.

The shape of the species-functional diversity relationship contains key information about the net increase in functional redundancy as a function of species diversity. However, it does not identify which function becomes redundant and if all functions behave the same way. Yet, these are crucial and unanswered questions. There are three possible scenarios: the redundancy of a given function can increase, decrease or remain stable when taxonomic diversity increases. It is important to realise that the three scenarios can co-occur within the same assemblage. Indeed, an increase in the functional redundancy of an assemblage only implies an increase in the ratio between the total number of functions and species. Within a function, redundancy can vary, as long as the net result across all functions is an increase. Identifying which function follows which scenario is crucial for understanding the future of an assemblage after a disturbance.

The study of functional redundancy requires a functional classification scheme, and deciding upon such scheme is not a neutral choice [21]–[23]. If every species is assigned a unique function, then the relationship between species and functional diversity will be linear with slope 1. In contrast, a gross classification scheme with only a few functions would quickly result in a flat asymptote. In between, when species are assigned to an intermediate number of functions, then the species-functional diversity relationship will have a slope of <1 and eventually reach an asymptote. Thus, virtually all of the theoretical scenarios in Figure 1 can be obtained just by using different classification schemes. At best, this is a limitation to studies aiming to investigate the shape of the relationship between species and functional diversity. At worst, this is a flaw that needs to be addressed. Multivariate methods that allow for continuous rather than discrete functional classifications have recently been developed [8], [21]. However, these methods remain sensitive to the initial traits analysed. In order to evaluate the sensitivity of results for the classification scheme used for functional groups, [16] proposed to examine the results for different combinations of functional traits, and thus for different classification schemes. Although this approach can improve our confidence on reported results, it does not explicitly test if the observed results are an artefact of the functional classification scheme used. Such a test would imply that the observed relationship between species and functional diversity is compared with a randomly generated relationship that would be obtained by chance only with the same functional classification scheme. Further to methodological issues, significant differences between observed and randomly generated relationships would imply that some functions are more important than others in the assemblage. Identifying these essential functions and their assembly rules would have important implications for both ecology and conservation.

Micheli and Halpern [16] provided one of the first studies of the relationship between species and functional diversity. However, they could not fully test alternative relationships as they only had datasets for low diversity assemblages. Furthermore, the role of habitat on this relationship was not considered and, to the best of our knowledge, no study has ever examined how within function redundancy varies with taxonomic diversity. Here, we surveyed coral reef fishes from a large Pacific island, New Caledonia, and provide, for the first time, a species-functional diversity relationship for one of the most diverse vertebrate assemblage on earth (New Caledonia comprises over 1700 reef fish species, [24]). First, we tested which theoretical model best fitted the observed relationship. This was done for three different functional classification schemes and comparisons were made between the observed and randomly generated relationships. Second, we examined redundancy within each functional group as a function of species richness. Last, we studied if habitat had an effect on the relationship between species and functional diversity, as well as on within function redundancy, a particularly relevant question in the context of the “coral reef crisis”, where phase shifts in coral reef dynamics are reported in leading journals [25].

## Materials and Methods

### Study site and data collection

**Figure 2:**
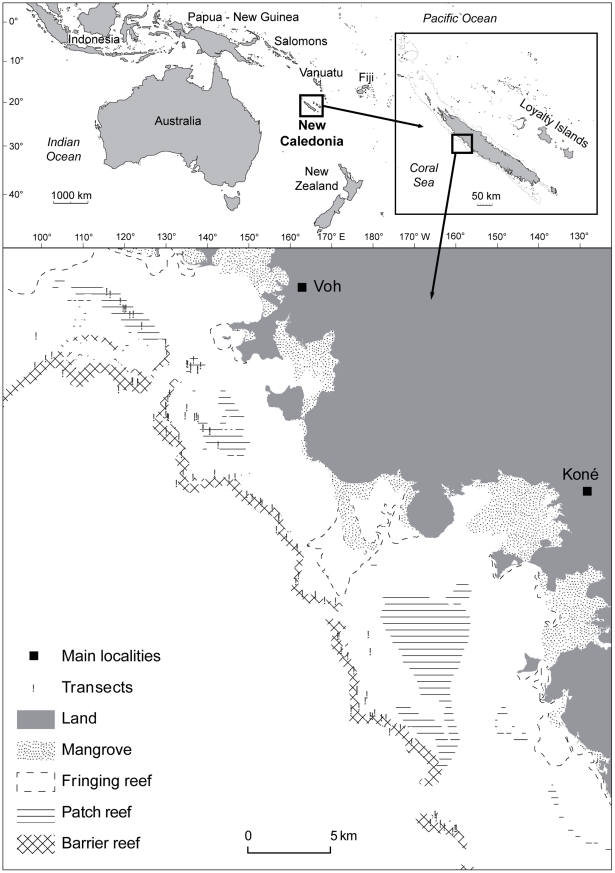
Location of the transects surveyed near Koné, north-western coast of New Caledonia, South-West Pacific. Reef types (from [29]), mangroves and land are indicated.

The study site was located on the north-western coast of New Caledonia, South Pacific (Figure 2). Fish and habitats were surveyed along 152 transects randomly distributed on patch reefs and inner barrier reefs, between 2002 and 2004. The transects were 50 m long and covered shallow reef areas.

Fish were surveyed using the distance sampling method: two divers swam side by side along each transect and recorded the number, fork length, and distance to transect of all visible fish [26], [27]. Possible observer effects were already examined in a previous study using the same dataset and showed no significant observer bias [28]. This study also underlined the remarkable stability of the functional structure of fish assemblages during the survey period, despite some natural disturbance [29], suggesting that the temporal fluctuations of the environment that occurred in this area were not an obstacle to the study of functional patterns [28].

Habitat was described along each transect using the “Medium Scale Approach” (MSA, [30]). Briefly, this method consisted of estimating the depth, habitat complexity (1: low, 2: medium-low; 3: medium-high; 4: high) and percentage cover of 14 substrate components (Table 1) in each of twenty 5×5 m quadrats distributed along each transect (10 quadrats on each side). Aggregated habitat variables were then built by grouping some of the substrate components into larger categories: live corals, hard bottom, soft bottom and coral shelter (Table 1). Mean depth, mean habitat complexity and substrate diversity (number of substrate components observed) per transect were calculated. At a larger scale, broad geomorphological reef types (barrier and patch reefs) were considered [31].

**Table 1:** Substrate elements for which percentage cover was estimated using the medium scale approach [30].

Non-living components	Living components
Mud (SB)	Encrusting corals (HB, LC)
Sand and gravel (SB)	Massive corals (HB, LC)
Debris *(piece of rock or debris less than 5 cm in its largest dimension)* (SB)	Branched corals (HB, LC, CS)
Small blocks *(piece of rock or dead coral 5–30 cm in its largest dimension)* (HB)	Digitate corals (HB, LC, CS)
Large blocks *(piece of rock or dead coral 30–100 cm in its largest dimension)* (HB)	Tabular corals (HB, LC, CS)
Rock (HB)	Foliose corals (HB, LC, CS)
Dead coral *(coral skeletons still in place)* (HB)	*Millepora* Corals (HB, LC, CS)

The substrate elements were grouped into the following categories: LC: live hard corals; HB: hard bottom; SB: soft bottom; CS: coral shelter.

Coral reefs from New Caledonia (covering ∼24 000 km^2^, among which ∼15 000 km^2^ were recently registered in the UNESCO World Heritage List) are one of the most pristine coral reef ecosystems in the world. Moreover, the study site was located in a rural area of New Caledonia, and could thus be considered as very well preserved from human influence, with regard to most of the large reef ecosystems in the world. In this respect, our dataset appears highly relevant to study patterns of functional structure and redundancy [32]. However, small-scale fishing activities occur in this area (mostly subsistence and recreational fisheries, resulting in relatively low levels of fishing pressure), which might impact the redundancy patterns of some functional groups [33]. Using spatial data from [33], each transect could be attributed a level of fishing pressure. It ranged from 0.01 to 3.2 t/km^2^/year, resulting in three categories: low fishing pressure (<0.5 t/km^2^/year), medium fishing pressure (0.5–1 t/km^2^/year), and high fishing pressure (>1 t/km^2^/year).

### Building functional groups

Three functional classification schemes were built by combining four functional traits: diet (P: piscivores, C: carnivores, H: herbivores-detritus feeders, Z: plankton feeders), adult size (1: <8 cm, 2: 8–15 cm, 3: 15–30 cm, 4: 30–50 cm, 5: 50–80 cm, 6: >80 cm), home range (1: sedentary; 2: mobile; 3: very mobile) and gregariousness (1: solitary; 2: paired; 3: small schools of 3–25 fish; 4: medium schools of 25–50 fish; 5: large schools of more than 50 fish). These traits have been defined elsewhere [28] and were retrieved from FISHBASE [34], FISHEYE [35], and [36]. Detailed traits for each observed species are provided in Table S1. The three functional classification schemes were:

- DS: diet (4 classes)×size (6), with 24 possible functions;

- DSH: diet (4)×size (6)×home range (3), with 72 possible functions;

- DSHG: diet (4)×size (6)×home range (3) × gregariousness (5), with 360 possible functions.

For convenience, the functions are hereafter coded with a letter (diet class) followed by numbers (size class, home range class and gregariousness class). For instance, “H3” corresponds to herbivores of size-class 3 (15–30 cm) and “C631” to very mobile >80 cm solitary carnivores.

### Data analyses

All analyses were repeated for each of the three functional classification schemes.

#### Functional-species diversity relationship

In order to determine the shape of the relationship between functional and species diversity, we fitted, by regression, linear (1), power (2), asymptotic (3), and logistic (4) through origin models to our dataset:
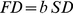
(1)

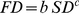
(2)

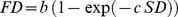
(3)

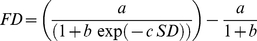
(4)


where FD = functional diversity (number of functions per transect), SD = species diversity (number of species per transect), and a, b, c = model parameters. Models were compared on the basis of percentage explained variance (*R^2^*), negative log-likelihood (NLL), and information criteria (Akaïke AIC and Bayesian BIC). Greater R^2^ and smaller NLL, AIC and BIC indicate a better fit. Nested models (linear versus power; asymptotic versus logistic) were formally compared by log-likelihood ratio tests [37]. These analyses were performed with R software®.

To test if the observed functional-species diversity relationships were an artefact of the functional classification schemes, we compared our observations with randomly generated relationships obtained from the same schemes. This was achieved by a Monte-Carlo analysis with 999 random permutations, using R®. At each permutation, each species was assigned a new function by randomly sampling without replacing the vector containing species' functional memberships. The random functional classification of species was then used to calculate the functional diversity of each transect. After 999 random permutations plus one observation, we obtained a set of 1000 functional-species diversity relationships. These formed a distribution from which the probability of the observed relationship could be determined.

The effect of habitat on the relationship between functional and species diversity was tested as follows. First, the transects were clustered by the chi square distance and Ward's minimum variance algorithms [38], [39] according to the grouped substrate categories (Table 1), depth, substrate diversity and habitat complexity. This was done for each reef type. Second, the functional-species diversity relationship was plotted and modelled for each habitat cluster using the most relevant model (identified from previous analyses), using Statistica®. A covariance analysis (ANCOVA) was then conducted to determine if the functional-species diversity relationships were significantly different between clusters using R®.

To ensure that the small-scale fishing activities occurring in this area did not influence the study of the functional-species diversity relationship, the same analyses as for the habitat clusters were conducted using the three levels of fishing pressure (i.e. plot of the functional-species diversity relationship for each fishing pressure category, and ANCOVA).

#### Functional redundancy

For each function, a Spearman's rank correlation analysis was conducted and a linear regression was fitted to examine whether the redundancy of each function increased, decreased or remained stable as species diversity increased. When a significant relationship was identified, least-square curves were fitted to visually assess its shape. These analyses were performed with Statistica®.

Generalized linear models (GLM) were used to examine the influence of habitat (reef type, grouped substrate categories, depth, substrate diversity and habitat complexity) on the redundancy of each function, using R®. This analysis was also performed for the functional redundancy of the whole assemblage. The same GLMs were performed a second time with an additional parameter: fishing pressure (categories described previously), to examine if this parameter influenced the redundancy of functions and the results obtained previously.

## Results

### Functional-species diversity relationship

#### Model comparison

**Figure 3:**
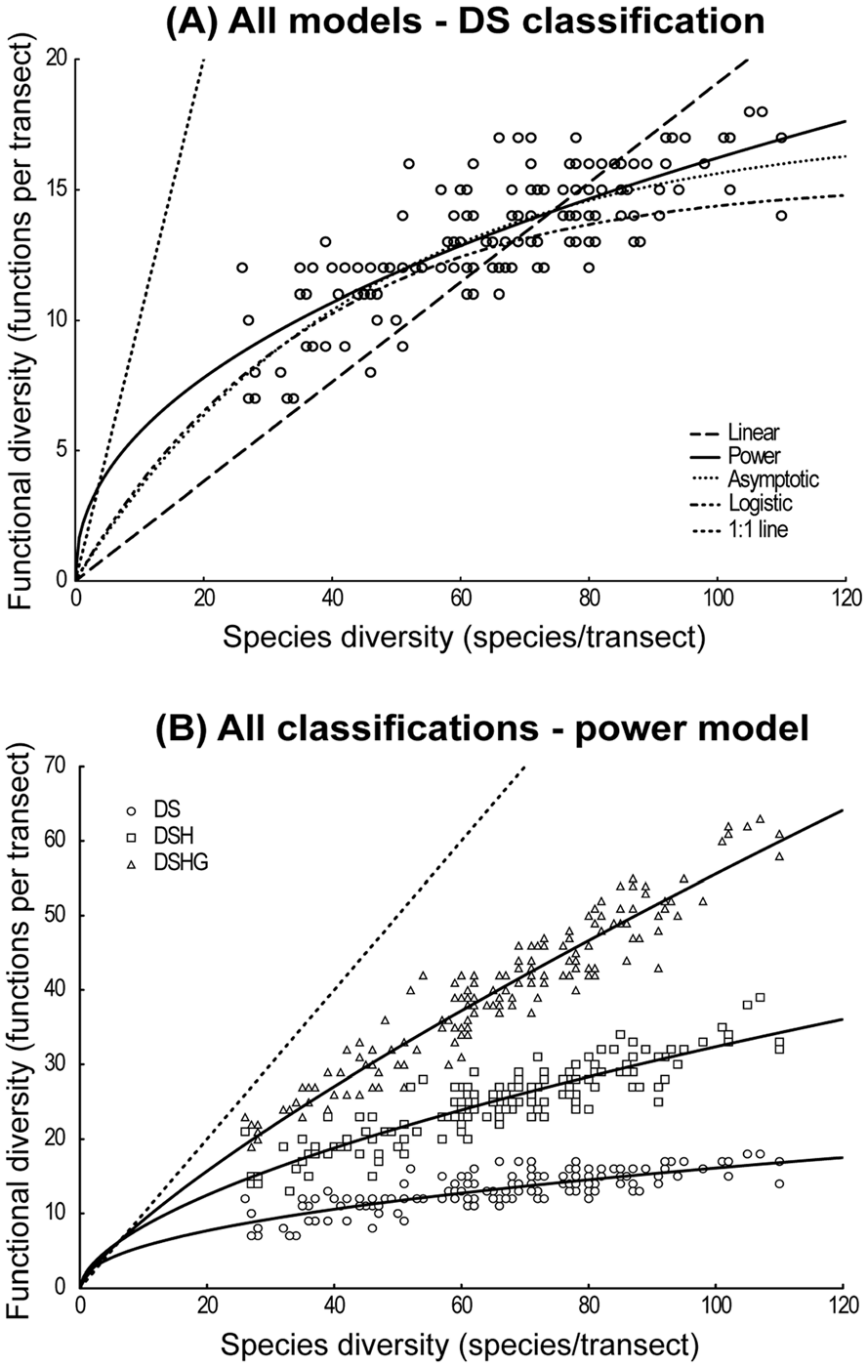
Relationship between species diversity (number of species/transect) and functional diversity (number of functions/transect). A: Linear, power, asymptotic and logistic regressions are shown for the DS classification scheme. B: only the best model (power, see Table 2) is shown for each of the three classification schemes. Dotted line indicates 1∶1 line for both plots. DS: diet×size; DSH: diet×size×home range; DSHG: diet×size×home range×gregariousness.

A total of 421 species belonging to 142 genera and 47 families were observed in the study area. These corresponded to 20 observed functions for the DS classification scheme, 46 for DSH, and 96 for DSHG. On a single transect, between 27 and 112 species corresponding to 7–18 (DS), 13–39 (DSH) and 19–63 (DSHG) functions were observed. For each classification, there was a significant (*P*<0.05) and positive correlation between the number of functions and the number of species (Spearman *R* = 0.76, 0.88 and 0.95 for DS, DSH and DSHG, respectively). Consistently across classification schemes, the relationships between functional and species diversity were non-linear (log-likelihood ratio tests, *P*<0.001 for all linear versus power model comparisons) and best described by a power model (Table 2, Figure 3).

**Table 2:** Results of the regression relationships between functional diversity and species diversity for the linear, power, asymptotic, logistic and through the origin models for each functional classification scheme (DS, DSH and DSHG).

	DS				DSH				DSHG			
Model	*R^2^*	NLL	AIC	BIC	*R^2^*	NLL	AIC	BIC	*R^2^*	NLL	AIC	BIC
Linear	0*	352	709	715	0.51	410	824	830	0.85	418	840	847
*Power*	*0.62*	*276*	*558*	*567*	*0.80*	*341*	*689*	*698*	*0.91*	*384*	*774*	*783*
Asymptotic	0.61	277	561	570	0.78	347	701	710	0.90	389	784	793
Logistic	0.61	295	598	610	0.78	428	864	876	0.90	421	850	862

DS: diet × size; DSH: diet × size × home range; DSHG: diet × size × home range × gregariousness.

*R^2^*: % variance explained by the models (least square fit), NLL: negative log-likelihood, AIC: Akaïke information criterion, BIC: Bayesian information criterion.

Larger *R^2^*, smaller NLL, AIC, BIC indicate better fit (italic).

*: Note that linear through origin models can have a negative *R^2^* when the variation around the regression line is greater than the variation around the mean. This occurred for the DS classification for which we set *R^2^* = 0 (rather than negative) to follow the usual convention. In view of this, the log-likelihood methods (NLL, AIC, BIC) were preferable to least-square fits for model comparisons.

Although asymptotic and logistic models provided a very similar fit to the data with regard to the power model, there was no visual (Figure 3) nor statistical evidence of an asymptote (Table 2). Rather, the power model indicated a rate of increase in functional diversity that was close to one until about 5–10 species, after which it declined but never went flat (Figure 3). There was no evidence of S-shaped relationships (Figure 3). For all classification schemes, the logistic model explained exactly the same amount of variance as the asymptotic model (Table 2), but with one more parameter. The log-likelihood ratio tests showed that the logistic model was indeed over-parameterized (*P*<0.001 for all asymptotic versus logistic model comparisons).

#### Effect of the functional classification schemes

**Figure 4:**
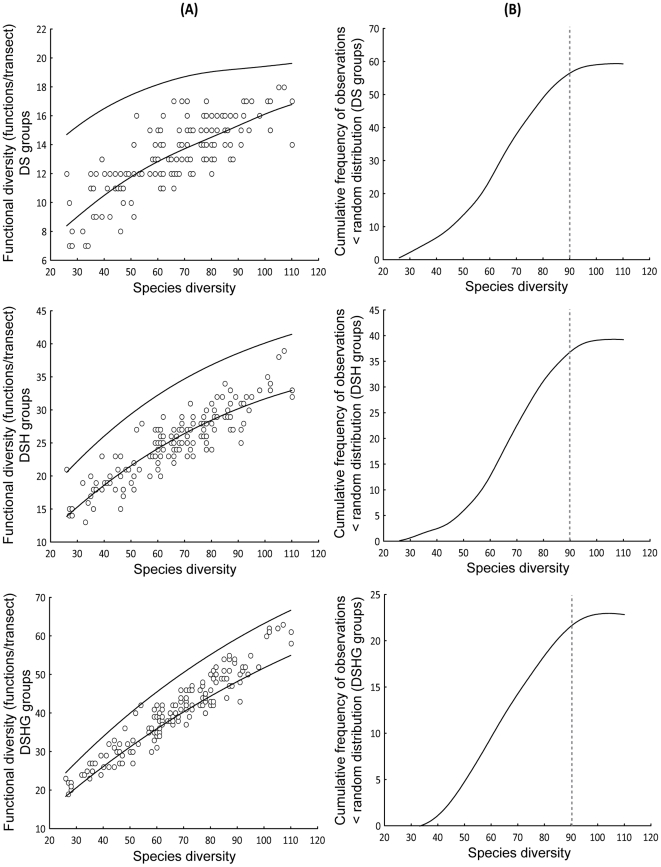
Comparing observed and random distributions of functions among species. A: Functional diversity (number of functions/transect) as a function of species diversity (number of species/transect) for the three functional classification schemes (DS, DSH, DSHG). Lines indicate 95% confidence intervals estimated from random distribution (Monte Carlo analysis with 999 permutations). B: Cumulative frequency distributions of observations for which the number of observed functions was significantly lower than the number of functions obtained from a random distribution (i.e. lower than the 95% confidence interval). DS: diet×size; DSH: diet×size×home range; DSHG: diet×size×home range×gregariousness.

Increasing the complexity of the functional classification scheme rendered the relationship between functional and species diversity more linear and closer to the 1∶1 line (Figure 3B). If we had chosen the possibly most complex classification where each species would represent a unique function, then observed and randomly generated functional-species diversity relationships would all have followed the same 1∶1 line, and thus would not differ. To some extent, this was observed for the DSHG classification where most observations (78%) fell within the 95% confidence interval estimated from 999 randomly generated relationships (Figure 4). However, none of the 22% of the DSHG observations that fell outside the 95% CI were greater than the upper limit of the CI (Figure 4A); this was true for all classification schemes. Moreover, the number of observations that were smaller than the 95% CI lower limit increased for simpler classifications, for which the general functional-species diversity relationship was therefore further from the 1∶1 line (Figure 4). Whereas 22% of the DSHG observations fell below the 95% CI, the percentage increased to 40% for DSH and 60% for DS (Figure 4B). Thus, there were significantly fewer functions in the observed assemblage than was expected by chance, implying that increasing species diversity primarily increased the redundancy of some essential functions rather than the functional diversity per se. Observations that fell below the 95% CI were not randomly distributed. Cumulative frequency distributions were S-shaped (Figure 4B), implying that past a certain level of species diversity, most observations fell within the CI (Figure 4A). Interestingly, this threshold was similar for all functional classification schemes and could be visually estimated at approximately 90 species (Figure 4).

#### Effect of habitat and fishing pressure

**Figure 5:**
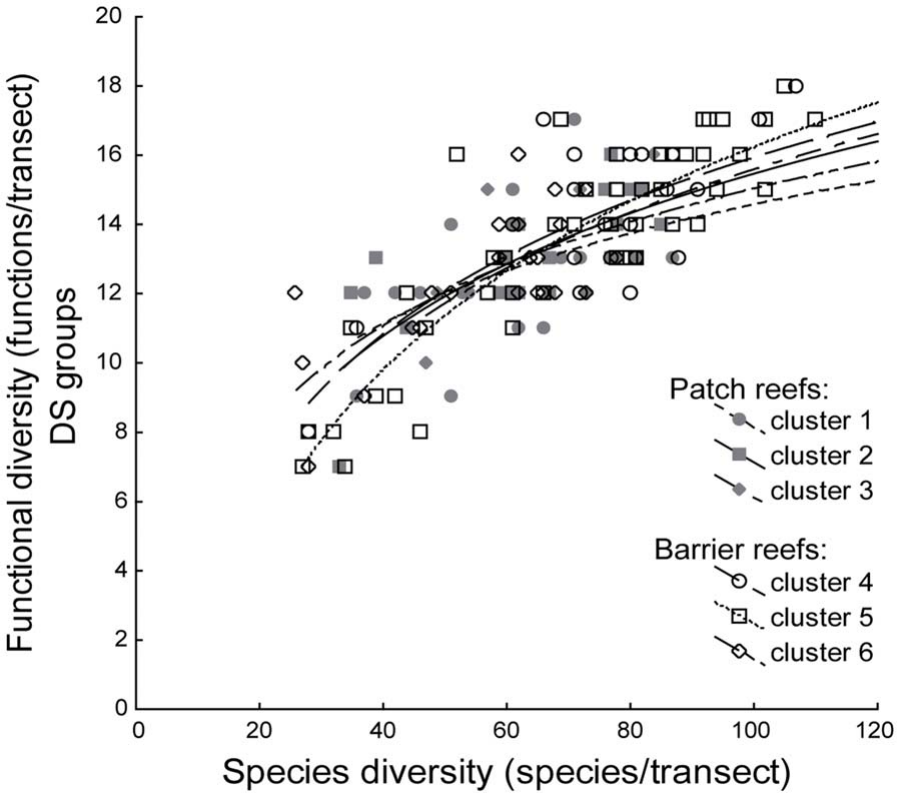
Relationship between species diversity and functional diversity for different types of reef habitats. Functional diversity (number of functions/transect, DS functional classification scheme) as a function of species diversity (number of species/transect) for each of the six clusters (three per reef type) obtained after the hierarchical classifications of transects according to their transect-scale habitat characteristics (see Table 3). DS: diet×size.

For each reef type, the transects could be classified into three distinct habitat clusters (Table 3). On barrier reefs, cluster 1 grouped transects with a high percentage of live corals, coral shelters, and hard bottoms, high habitat diversity and complexity, and a low percentage of soft bottoms. Cluster 3 contained transects with the opposite characteristics, notably with a high percentage of soft bottoms, and cluster 2 displayed intermediate values (Table 3). The clustering was similar on patch reefs. Habitat cluster 4 was dominated by soft bottoms, cluster 6 by hard substrate components and cluster 5 was characterized by fairly similar proportions of soft and hard substrates (Table 3). Despite these distinct habitat characteristics, the relationships between functional and species diversity were not significantly different between the six habitat clusters (ANCOVA, *P*>0.05 for all functional classification schemes, Figure 5).

**Table 3:** Description of the clusters obtained after the hierarchical classification of transects according to their transect-scale habitat characteristics, for two reef types.

Reef type	Habitat cluster	% Live corals	% Coral shelter	% Hard bottom	% Soft bottom	Habitat diversity	Depth	Habitat complexity
Barrier reefs	1	13.7 (8.2)	10.1 (12.3)	57.7 (12.9)	30.1 (9.5)	13.5 (2.4)	1.7 (0.5)	2.1 (0.6)
	2	12.2 (7.7)	6.6 (5.9)	40.3 (6.4)	56.1 (6.1)	12.8 (1.8)	1.9 (0.8)	1.7 (0.4)
	3	6.2 (3.9)	3.5 (2.6)	18.4 (6.8)	75.3 (9.2)	11.7 (2.8)	1.5 (0.8)	1.5 (0.3)
Patch reefs	4	7.8 (5.7)	4.2 (4.4)	31 (11.3)	62.6 (9.5)	13.5 (3.1)	2.6 (1)	1.5 (0.5)
	5	18.7 (11.6)	6.1 (5.6)	51.4 (4.2)	44.9 (3.8)	13.6 (2.8)	3.1 (0.8)	2.0 (0.5)
	6	25.4 (10.7)	6.5 (3.6)	67.7 (8.2)	22.8 (5.2)	13.8 (1.7)	3.0 (1.1)	2.3 (0.3)

The average value per cluster is given for each habitat variable (standard deviation is indicated in parentheses).

**Figure 6:**
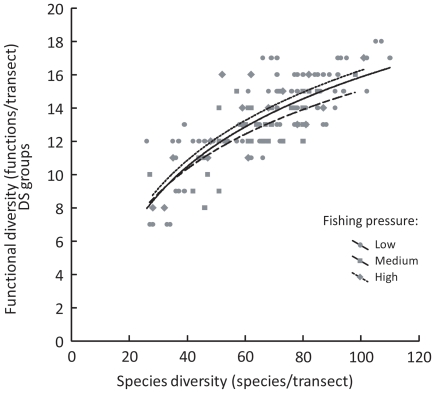
Relationship between species diversity and functional diversity for different levels of fishing pressure. Functional diversity (number of functions/transect, DS functional classification scheme) as a function of species diversity (number of species/transect) for each of the three categories of fishing pressure (derived from [33]). DS: diet×size.

Similarly, the relationships between functional and species diversity were not significantly different between the three categories of fishing pressure (ANCOVA, *P*>0.05 for all functional classification schemes, Figure 6), suggesting little or no effect of a weak fishing pressure on our conclusions.

### Functional redundancy

#### Relationship with species diversity

The distribution of species among functions showed that a large proportion of species belonged to only a few functions (Table 4). For the DS classification scheme, three functions (C3, C2 and H2) out of 20 comprised ∼50% of species on average (and seven functions represented ∼83% of species). Similar distribution patterns were obtained for DSH and DSHG classification schemes.

**Table 4:** Average number of species per function (functional redundancy) and average proportion of species diversity (functional dominance) for the diet × size (DS) classification scheme.

Function (DS)	Average number of species (Std Dev.)	Average proportion of species diversity (%)	Cumulative number of species	Cumulative proportion (%)
C3	16.9 (0.9)	25.6	16.9	25.6
C2	9.4 (3.1)	14.2	26.3	39.8
H2	7.6 (5.3)	11.6	33.9	51.4
H3	5.6 (2.8)	8.5	39.5	59.9
Z2	5.2 (1.4)	7.9	44.7	67.8
C4	5.1 (0.7)	7.8	49.8	75.6
H4	5 (3.1)	7.6	54.8	83.2
H5	2.9 (2.3)	4.3	57.7	87.5
C5	2.2 (2.3)	3.3	59.9	90.8
Z1	1.1 (1.6)	1.7	61	92.5
C1	1.1 (0.3)	1.6	62.1	94.1
P4	1 (0.6)	1.5	63.1	95.6
Z3	0.9 (0.8)	1.4	64	97.0
P3	0.6 (1)	0.9	64.6	97.9
C6	0.4 (0.5)	0.6	65	98.5
P2	0.3 (0.5)	0.5	65.3	99.0
P5	0.3 (0.7)	0.4	65.6	99.4
P6	0.2 (2.6)	0.4	65.8	99.8
P1	0.1 (0.9)	0.1	65.9	99.9
Z4	0.1 (0.3)	0.1	66.0	100.0

**Figure 7:**
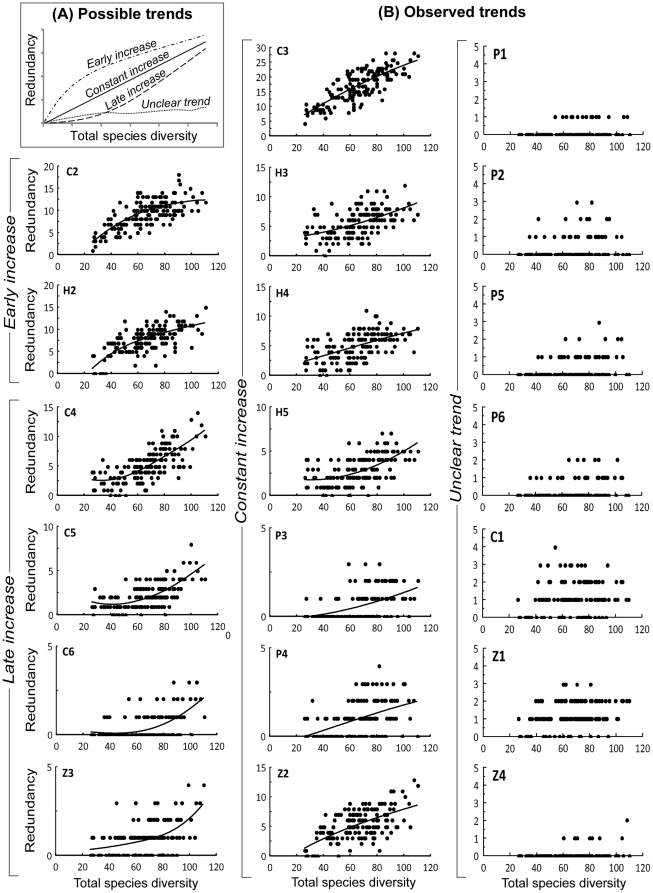
Redundancy as a function of species diversity, for individual functions (DS functional classification scheme). A: Schematic possible trends. B: Observed trends. The plots were classified as early increase, late increase, constant increase or unclear trend (see Table 5). Least-square curves were fitted to each plot (for which the slope was significant following a linear regression) to visualise the trends. DS: diet×size.

There was a significantly positive correlation between functional redundancy and species diversity for all 20 functions of the DS classification scheme (Spearman's correlation tests, *P*>0.05), indicating that redundancy was generally higher in diverse assemblages. However, the slope of the relationship was significantly positive for only 13 functions (Table 5). Among these, three patterns of increase could be identified (Figure 7). First, the redundancy of some functions increased rapidly at low levels of species diversity, and then slowed down at higher levels. For instance, the redundancy of C2 and H2 increased notably between 0 and approximately 60 species, and then increased with a gentler slope past this limit (Figure 7). Second, some functions displayed slow increases of redundancy at low levels of species diversity, and faster increases at higher levels; C4 and C5 provided good examples of this pattern, with respective thresholds of approximately 50 and 70 species (Figure 7). Third, some functions, such as C3 and H4, displayed a linear pattern of increase of their redundancy as a function of species diversity (Figure 7). The remaining functions displayed unclear trends (supported by non-significant slopes and low Spearman's correlation coefficients with regard to the other functions) and corresponded to low levels of functional redundancy (Table 5, Figure 7).

**Table 5:** Spearman's rank correlation coefficients and slopes of linear regressions between the redundancy of a function (DS functional classification scheme) and species diversity.

Function (DS)	Correlation coefficient (Spearman)	Slope of linear regressions
C1	0.21 **	ns
C2	0.64 ***	0.15 ***
C3	0.76 ***	0.22 ***
C4	0.72 ***	0.11 ***
C5	0.61 ***	0.05 **
C6	0.46 ***	0.02 **
H2	0.66 ***	0.11 *
H3	0.57 ***	0.07 ***
H4	0.58 ***	0.07 *
H5	0.58 ***	0.05 *
P1	0.2 *	ns
P2	0.29 ***	ns
P3	0.49 ***	0.02 *
P4	0.48 ***	0.02 *
P5	0.23 **	ns
P6	0.22 **	ns
Z1	0.28 **	ns
Z2	0.52 ***	0.09 *
Z3	0.41 ***	0.02*
Z4	0.19 **	ns

The levels of significance (*P*-values) are indicated for both Spearman's correlations and linear regressions. ns: not significant; *: *P*<0.05; **: *P*<0.01; ***: *P*<0.001; DS: diet×size.

The patterns observed for the DS classification schemes were also observed when considering the more complex DSH and DSHG functional classification schemes.

#### Effect of habitat

The GLMs indicated that habitat had little influence on the redundancy of functions. For the DS classification scheme (Table S2), the *R^2^* of the models ranged from 0.001 to 0.25 (mean: 0.11), and among the 168 possible functions×habitat effects, only 37 were significant. Two functions (P2 and Z4) were not influenced by any of the tested habitat factors. Most functions (14/20) were influenced by three or less habitat factors (out of eight), except H4, which showed significant effects for four habitat factors. None of the habitat factors had an effect on the redundancy of the whole assemblage, except for habitat diversity (Table S2). Similar results were obtained for the other functional classification schemes (DSH and DSHG).

#### Effect of fishing pressure and relevance of the dataset

**Figure 8:**
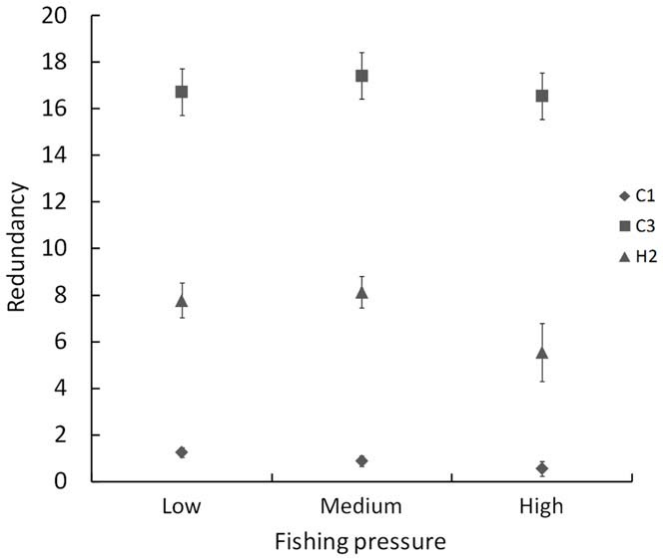
Average redundancy of three functions, for different levels of fishing pressure. Average redundancy of the three functions which showed a significant response to fishing pressure in GLMs (C1, C3 and H2), for each category of fishing pressure. Confidence intervals (95%) are indicated.

When adding fishing pressure in the analyses, the *R^2^* of the models were not improved (minimum: 0.005; maximum: 0.28; mean: 0.11), and most functions (17/20) were not significantly influenced by this factor (Table S3). C1, C3 and H2 were the only functions which redundancy responded significantly to fishing pressure. However, these responses appeared to be weak, as shown in Figure 8, again suggesting little or no effect of a weak fishing pressure on our conclusions. Similar results were obtained for the other functional classification schemes (DSH and DSHG).

## Discussion

### Functional-species diversity relationship

In order to address the issues regarding the extirpation or extinction of species and their consequences on the functioning of ecosystems, ecological research has witnessed increased interest in the patterns linking biodiversity with ecosystem functioning and processes [2], [7]. In this rising interest in functional ecology as a means of understanding the mechanisms structuring living assemblages, the general shape of the relationship between functional diversity and species diversity has often been discussed [40]–[44]. However, most work has focused on terrestrial ecosystems and only a few studies have dealt with marine environments [6], [16], [45]–[47]. Some propositions were made regarding the plausible shape of this relationship for coastal marine assemblages [16], [46], yet these were rarely illustrated with empirical validation [47], especially when dealing with species-rich communities like coral reef fish. The dataset used in this paper brings novel and essential clues regarding the general shape of this relationship in one of the most pristine and diversified ecosystems.

#### Non-random allocation of species to functions

Our results indicated that coral reef fish assemblages in New Caledonia functioned with a significantly lower number of functional groups than expected by chance. This suggests that the fulfilled functions were not a random selection of the possible functions. In an assemblage, this implies that certain functions would preferentially exist and that an increase in species diversity would result in an increase in the redundancy of these functions rather than generate new functions. However, this does not necessarily mean that the species fulfilling these preferential functions would be the same for all assemblages. Indeed, their identity could vary according to environmental conditions, habitat and regions. Petchey and Gaston [48] also reported a non-random distribution of species amongst the available functions in bird assemblages. More recently, Halpern and Floeter [47] reported this phenomenon for Atlantic reef fishes. Their study was conducted at the macro-scale, with each observation being a species list in a different site of the tropical Atlantic, and which involved fish assemblages much less diversified than those in New Caledonia. Our study revealed, for the first time, that the structured allocation of species to functions also occurs at a local scale, implying that the assembly rules observed at the macro-scale in the Atlantic could be derived from local scale rules. Furthermore, we showed for the first time that this phenomenon was only observed for low to intermediate levels of species diversity. Indeed, past a certain limit, estimated at ∼90 species/transect in the present case, the number of observed functions was not significantly different from a random situation. It is likely that such a changing pattern in the allocation of species to functions and its link with the level of species diversity may not be detectable unless contrasting levels of species diversity, and highly diverse assemblages such as those of New Caledonia, are considered.

Regarding the functional structure of fish assemblages on the scale of a reef, our results allow the formulation of a hypothesis which states that the allocation of species to functions is focused on a few functions when the “first species” of an assemblage are added. This remains true for intermediate levels of species diversity. This phenomenon might be linked to the existence of primordial functions that would be fulfilled in priority for an assemblage to gain its basic ecological structure. Such functions could constitute the functional core of assemblages and would become redundant very early, securing their resistance to disturbances and thus the resilience of the assemblage [4], [49]. Once these functions are fulfilled and sufficiently redundant, the addition of new functions would become less structured and may tend towards a random allocation of species to the remaining possible functions. The latter functions could be non-compulsory functions in simple assemblages, thus being secondarily fulfilled and only in especially rich assemblages. It is noteworthy that the level of species diversity necessary for the random creation of functions was high in the present study (90 species). This can be linked with several works that evoked the necessity of high species diversity and high functional redundancy of some essential functions to ensure the resistance and resilience of reef assemblages [4], [25], [50]–[55].

However, it is noteworthy to mention that functional redundancy is not the only parameter to contribute to the stability of assemblages. In particular, several studies highlighted the importance of the response diversity of an assemblage in the case of a disturbance [4], [56]. Indeed, differences in the response of the species composing a functional group also strongly contribute to the potential persistence of this group in the ecosystem. If a disturbance impacts all the constituent species of a functional group in the same way (e.g. fishing gear based on a particular functional trait, like gillnets), redundancy may be useless in improving the resistance of this function. Rather, the response traits of each of these species may be more important in this particular case [57]. Although redundancy plays an important role in ecosystem functioning, as shown in this paper, other processes are likely at stake to determine the resistance and resilience of an assemblage confronted with a disturbance.

#### Shape of the functional-species diversity curves

As in other studies, our results showed that the relationship between functional diversity and species diversity increased, going through the origin, and that it displayed an incremental decreasing rate when species diversity increased. This general shape was similar for the different functional classification schemes (DS, DSH and DSHG), even though the curve of the relationship differed according to the classification. Thus, scenario C from Micheli and Halpern [16] appeared to be the most relevant theoretical curve in the present study (Figure 1). For the first time, however, we were able to analyse the functional-species diversity relationship in the case of a highly diversified assemblage, and we could not detect any flat asymptote. Working on even more diversified assemblages may allow the likeliness of an asymptotic relationship to be examined. However, although the increasing rate of functional diversity slowed down for high levels of species diversity, it seems most unlikely that a strictly flat asymptote (i.e. no further creation of functions as species diversity increases) would be observed in real assemblages, except when using an oversimplified functional classification scheme. This is important for conservation ecology as this result suggests that some functions, most likely rare functions fulfilled by rare species, can only occur at the highest diversities.

The approach used for building our functional classification schemes and the comparison with random models improved our confidence in the reported results. Nevertheless, some improvements are still necessary in defining the functions. For example, the theoretical curves presented in [16] correspond to situations where either one function corresponds to one species (scenario A1) or one function corresponds to several species (scenario A2, B and C). The situation where one species corresponds to several functions has not been considered. Yet, in a real assemblage, it is known that a given species may change functions during its life history. The most common case is a change in the functional role of the species between juvenile and adult stages [36], [58]–[59], with juveniles sometimes fulfilling successive and distinct functions. More rarely, some changes in functions have been observed in adult populations, either through the appearance of sleeping functions (i.e. a change in the functional role of a species in response to a change in its environment or ecosystem) [60] or through multiple functions for the same species, but different morphs [61]. Thus, it is probable that the exact shape of the functional-species diversity curves would be different if the functional roles of juveniles were considered and/or if functional traits of species were better known. This underlines the necessity of gathering more complete biological and ecological data on reef fish species, among which certain species still remain poorly documented [62], [63].

### Functional redundancy and the level of species diversity

Beyond the general functional-species relationship, the consideration of each function and its redundancy may give further insights about the influence of species diversity on the functional structure of assemblages. Our results showed that, on average, high functional redundancy was only restricted to a few functions (Table 4, Figure 7). The corollary, a low redundancy in the majority of functions, has often been reported in coastal marine assemblages [16], [64], [65]. This means that the distribution of species among functions is very unbalanced. As a result, many low redundancy functions are likely to be empty on a single transect, suggesting the existence of rare functions only in certain locations or at a certain spatial scale [9], [60]. Such a large number of poorly redundant functions also suggest that the removal of certain species could correspond to the removal of a whole function, even in a species-rich assemblage, with important consequences on the assemblage's functional balance [9], [66], [67]. Large piscivores (P5 and P6 in the present study), which are more likely to be targeted by fishermen and which could disturb a top-down driven ecosystem if removed, illustrate such a situation [68], [69]. However, although the low redundancy of a function can be related to its vulnerability to species-removing disturbances, the demography and the abundance characterizing the species that fulfil this particular function are to be considered as well. As mentioned before, so is the diversity of the response traits of the species composing the function [4], [56], [57].

Our results also showed that the redundancy of a given function may vary with the level of species diversity, following four patterns. The first pattern was well illustrated by function C2, in which redundancy rapidly increased at low species diversities before stabilizing past an intermediate level of ∼60 species (Figure 7). A similar pattern was observed for functions C3 and H3 which were also, together with C2, among the most redundant functions, on average, in the assemblages (Table 4). A closer look at the composition of these three functions indicated that very few of their species were sedentary (Table S1). Such observations suggest a common core of medium sized herbivores and carnivores in a large variety of assemblages, even if their species compositions may partially vary from one location to the next. In this respect, the study of the redundancy of functions may point towards pioneer functions in assemblages, such as C2, C3 and H3 in the present case study. These results also suggest that these functions could be widespread and of similar importance to the majority of reef types and habitats, which would imply some constant features in the functioning of assemblages. Typically, because they are very dominant functions at low to intermediate species diversities, such functions may correspond to the essential functions suggested by the non-random allocation of species to functions (Figure 4). Thus, the structuring of assemblages around pioneer functions may be one of the key mechanisms explaining the non-random allocation of species to functions at low to intermediate species diversity levels. However, further studies are required to test this hypothesis.

The second observed pattern corresponded to functions for which redundancy only increased at high levels of species diversity. Functions C4 and C5 were representative of this pattern, being strongly redundant only past a threshold of ∼50 and ∼70 species, respectively. In contrast to pioneer functions (the first pattern), such functions may not exist at low levels of species diversity as their establishment requires a highly diverse assemblage. The example of C4 and C5 is typical, as large carnivores are often absent from species-poor assemblages where the diversity and abundance of prey may not be sufficient to sustain this functional niche [70], [71]. In the extreme, this pattern may represent rare functions that only exist in particularly diverse assemblages and/or in specific environments.

Third, some functions displayed an increase of redundancy following a constant incremental rate. All trophic groups and most of the size classes were represented among these functions (C6, H2, H4, H5, P3, P4, Z2 and Z3). Such functions may be ubiquist and therefore have a rather constant dominance in the different assemblages. Their role in the functional structure of assemblages would thus be similar regardless of the level of species diversity of these assemblages.

Lastly, seven functions showed unclear trends. These functions were present at most levels of species diversity, displaying a stagnant and low redundancy as species diversity increased (i.e. a decreasing dominance in assemblages).

### Effect of habitat on the functional structure of reef fish assemblages

Accounting for habitat factors and environmental contexts is crucial for understanding ecosystem functioning or the assembly rules of marine communities [1], [64], [72]. Yet, only a few studies examined the link between habitat and the functional structure of reef fish assemblages [73], [74]. Most focused on the correlations between environmental characteristics and the level of functional diversity. None dealt with species-rich reef fish assemblages, small-scale (transect) habitat factors, and the influence of habitat on functional redundancy. Yet, it is thought that changes in habitats, either due to natural variations or to anthropogenic disturbances, influence both the general pattern of the species-functional diversity relationship (scenario C, Figure 1) and the redundancy of functions.

We found that small-scale habitat factors had very little effect on the shape of the functional-species relationship (Figure 5). In this respect, scenario C (Figure 1) from [16], predicting a possible shift in the functional-species diversity curve when transitioning from one habitat to another, was not supported by our data. We also found few effects of habitat on the redundancy of functions. Most (78%) habitat factors had no significant effect on functional redundancy, and the correlations between habitat and redundancy were very poor for all functions. These results were surprising when considering that small-scale habitat factors are widely known to influence species composition, abundance and biomass in reef fish assemblages, as well as the abundance of some trophic groups [75]–[77]. For instance, species diversity is known to increase with coral cover, habitat complexity and hard substrate cover [78]–[80], and herbivores are generally less abundant when the algal cover is high [53], [81], [82]. Two alternative hypotheses may explain a functional similarity between different habitats despite some taxonomic dissimilarity. First, habitat may drive the local expression of the functional organization of an ecosystem by influencing the identity (and abundance) of the species locally comprising this organization, rather than the organization per se. In this respect, our results suggested that if the characteristics of the functional-species relationship were to be determined by environmental factors, larger-scale factors might be involved rather than small-scale factors. This could mean that some important aspects of the functional structure of reef fish assemblages would be independent from small-scale habitat features, as suggested by Bellwood and Hughes [50] for reef fishes in the Indo-Pacific. Second, connectivity between the habitats of an ecosystem may be sufficient to ensure some functional similarity. Although further studies are required to test these hypotheses, the functional similarity between different coral reef habitats indicates that they are part of the same functional unit, and should therefore be managed as such.

Species-rich ecosystems such as coral reefs are degrading at a fast rate due to a number of human-induced factors including fishing, global warming, and urbanization (e.g. [48]). Because little is known about the functioning of these ecosystems, their protection mainly involves the creation of MPA networks that are most often and very conveniently designed from habitat or species diversity maps (e.g. [83]), or with the lowest socio-economic costs (e.g. [84]). However, we have no idea if this approach can really ensure the normal functioning of ecosystems and thus their resilience to perturbations. Our study showed that the functional-species relationship for coral reef fish assemblages in New Caledonia was not asymptotic, implying that rare functions and species mainly occur in highly diverse assemblages. This calls for the protection of biodiversity hotspots. We also found that the assembly rules of these reef fish species were not random. When diversity increases, some functions are rapidly reinforced and others only appear at high diversity levels. This finding has direct implications in restoration ecology where the chronology of successive assemblages is crucial. Finally, we found functional similarity between the different habitats of the coral reef ecosystem, suggesting a single functional and thus management unit in this highly complex ecosystem. Our study provides novel information regarding the functional structure of species-rich fish assemblages, but further knowledge is required to be able to verify whether or not current management plans are adequately ensuring the normal functioning of species-rich ecosystems.

## Supporting Information

Table S1 **Observed reef fish species and corresponding functional traits, as used for building functional groups: diet (P: piscivores, C: carnivores, H: herbivores-detritus feeders, Z: plankton feeders), adult size (1: <8 cm, 2: 8–15 cm, 3: 15–30 cm, 4: 30–50 cm, 5: 50–80 cm, 6: >80 cm), home range (1: sedentary; 2: mobile; 3: very mobile) and gregariousness (1: solitary; 2: paired; 3: small schools of 3–25 fish; 4: medium schools of 25–50 fish; 5: large schools of more than 50 fish).**
(XLS)

Table S2 **GLM testing the effects of habitat on the redundancy of each observed function (diet×size classification scheme) and on the functional redundancy of the whole coral reef fish assemblage.**
(DOC)

Table S3 **GLM testing the effects of habitat and fishing pressure on the redundancy of each observed function (diet×size classification scheme) and on the functional redundancy of the whole coral reef fish assemblage.**
(DOC)
