# Supplementary material for: Functional Redundancy Patterns Reveal Non-Random Assembly Rules in a Species-Rich Marine Assemblage
Source: PLoS One. 2011 Oct 21;6(10):e26735. doi: 10.1371/journal.pone.0026735 (PMC3198825; doi:10.1371/journal.pone.0026735)
Supplement: Table S2 — GLM testing the effects of habitat on the redundancy of each observed function (diet×size classification scheme) and on the functional redundancy of the whole coral reef fish assemblage. (DOC) [file pone.0026735.s002.doc]

| Redundancy | | Model R² | Reef type effects | Transect-scale habitat effects | | | | | | |
| --- | --- | --- | --- | --- | --- | --- | --- | --- | --- | --- |
|  |  |  |  | % Live corals | % Coral shelter | % Hard bottom | % Soft bottom | Mean depth | Habitat diversity | Mean habitability |
| Assemblage | | 0,06 | NS | NS | NS | NS | NS | NS | P=0.037 (-) | NS |
| Function | C1 | 0,25 | P<0.001 (M) | NS | NS | NS | NS | P=0.002 (+) | NS | NS |
|  | C2 | 0,03 | NS | NS | NS | P=0.019 (+) | NS | NS | NS | NS |
|  | C3 | 0,2 | P<0.001 (B) | P=0.033 (+) | NS | P=0.011 (-) | NS | NS | NS | NS |
|  | C4 | 0,1 | P=0.025 (B) | P<0.001 (+) | NS | NS | NS | NS | NS | NS |
|  | C5 | 0,17 | P<0.001 (B) | P=0.012 (+) | NS | NS | NS | P=0.042 (+) | NS | NS |
|  | C6 | 0,09 | NS | NS | NS | P=0.038 (+) | P=0.022 (+) | NS | NS | NS |
|  | H2 | 0,05 | NS | NS | NS | P=0.032 (+) | NS | NS | P=0.006 (+) | NS |
|  | H3 | 0,03 | NS | P=0.004 (+) | NS | NS | NS | NS | NS | NS |
|  | H4 | 0,21 | NS | P=0.005 (-) | NS | P<0.001 (+) | NS | P<0.001 (+) | NS | P=0.037 (+) |
|  | H5 | 0,15 | NS | P=0.035 (-) | NS | NS | NS | P=0.005 (+) | NS | P<0.001 (+) |
|  | P1 | 0,02 | NS | NS | NS | NS | NS | NS | NS | P=0.013 (+) |
|  | P2 | 0,001 | NS | NS | NS | NS | NS | NS | NS | NS |
|  | P3 | 0,13 | P<0.001 (B) | NS | NS | NS | P=0.026 (-) | NS | NS | NS |
|  | P4 | 0,15 | P<0.001 (B) | NS | NS | NS | NS | NS | NS | NS |
|  | P5 | 0,01 | NS | P=0.038 (+) | NS | NS | NS | NS | NS | NS |
|  | P6 | 0,1 | P=0.019 (B) | P=0.004 (+) | NS | NS | NS | P=0.029 (+) | NS | NS |
|  | Z1 | 0,22 | P<0.001 (M) | NS | NS | NS | NS | P<0.001 (+) | P=0.037 (+) | NS |
|  | Z2 | 0,09 | NS | NS | NS | NS | NS | P=0.001 (+) | NS | NS |
|  | Z3 | 0,1 | NS | NS | NS | NS | NS | P=0.001 (+) | NS | NS |
|  | Z4 | 0,001 | NS | NS | NS | NS | NS | NS | NS | NS |

For significant effects, *P*-values and effect signs (in parentheses) are indicated. B: redundancy is higher on barrier reefs; M: redundancy is higher on patch reefs; +: redundancy increases when the habitat variable increases; -: redundancy decreases when the habitat variable increases; NS: not significant; DS: diet × size.
